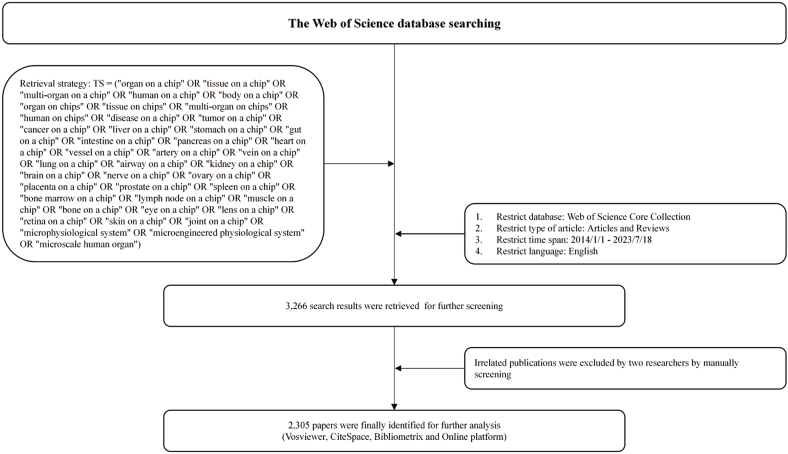# Emergent trends in organ-on-a-chip applications for investigating metastasis within tumor microenvironment: A comprehensive bibliometric analysis

**DOI:** 10.1016/j.heliyon.2023.e23504

**Published:** 2023-12-09

**Authors:** Chunrong He, Fangfang Lu, Yi Liu, Yuanhu Lei, Xiaoxu Wang, Ning Tang

**Affiliations:** a Department of Orthopaedics, The Second Affiliated Hospital, University of South China, Hengyang, Hunan, China; b Department of Ophthalmology, The Second Affiliated Hospital, University of South China, Hengyang, Hunan, China; c Department of Orthopaedics, The Third Xiangya Hospital of Central South University, Changsha, Hunan, China

**Keywords:** Organ-on-a-Chip, Tumor microenvironment, Metastasis, Bibliometric, Vosviewer

## Abstract

**Background:**

With the burgeoning advancements in disease modeling, drug development, and precision medicine, organ-on-a-chip has risen to the forefront of biomedical research. Specifically in tumor research, this technology has exhibited exceptional potential in elucidating the dynamics of metastasis within the tumor microenvironment. Recognizing the significance of this field, our study aims to provide a comprehensive bibliometric analysis of global scientific contributions related to organ-on-a-chip.

**Methods:**

Publications pertaining to organ-on-a-chip from 2014 to 2023 were retrieved at the Web of Science Core Collection database. Rigorous analyses of 2305 articles were conducted using tools including VOSviewer, CiteSpace, and R-bibliometrix.

**Results:**

Over the 10-year span, global publications exhibited a consistent uptrend, anticipating continued growth. The United States and China were identified as dominant contributors, characterized by strong collaborative networks and substantial research investments. Predominant institutions encompass Harvard University, MIT, and the Chinese Academy of Sciences. Leading figures in the domain, such as Dr. Donald Ingber and Dr. Yu Shrike Zhang, emerge as pivotal collaboration prospects. Lab on a Chip, Micromachines, and Frontiers in Bioengineering and Biotechnology were the principal publishing journals. Pertinent keywords encompassed Microfluidic, Microphysiological System, Tissue Engineering, Organoid, *In Vitro*, Drug Screening, Hydrogel, Tumor Microenvironment, and Bioprinting. Emerging research avenues were identified as "Tumor Microenvironment and Metastasis," "Application of organ-on-a-chip in drug discovery and testing" and "Advancements in personalized medicine applications".

**Conclusion:**

The organ-on-a-chip domain has demonstrated a transformative impact on understanding disease mechanisms and drug interactions, particularly within the tumor microenvironment. This bibliometric analysis underscores the ever-increasing importance of this field, guiding researchers and clinicians towards potential collaborative avenues and research directions.

## 1 Introduction

Organ-on-a-chip, a burgeoning area of bioengineering, represents a cutting-edge technology that has recently garnered substantial attention in the fields of, disease modeling, drug development and precision medicine [[1], [2], [3]]. These microfluidic cell culture devices, which can simulate the physiology and mechanics of entire organs or organ systems, offer unprecedented opportunities for scientific advancement and are expected to revolutionize traditional *in vitro* methodologies [1,4,5]. As of 2023, the organ-on-a-chip technology has been used to simulate a multitude of organs, including the heart, lung, liver, kidneys, and as well as the blood-brain barrier [3,4,[6], [7], [8], [9], [10]].

Pertaining to tumor researches, by creating personalized organ models that closely mimic the tumor microenvironment, organ-on-a-chip technology offers a more appropriate approach for individualized treatment plans [[11], [12], [13]]. By sourcing patient-derived cells for the chip-based systems, researchers can investigate the patient-specific mechanisms of metastasis and response to therapeutics [[14], [15], [16]]. This capability may lead to the development of personalized treatment strategies aimed at preventing or limiting metastasis.

However, despite the promising potential of these microphysiological systems, their development and standardization remain challenging, and their implementation in the pharmaceutical and healthcare industries is still in its infancy [17,18]. The heterogeneity of organ systems and the complexity of mimicking these on a microscale chip pose significant challenges that need to be overcome [2,19,20]. As with any new technology, there is also a steep learning curve, necessitating comprehensive training for researchers in fabrication, operation, and analysis of these devices.

In this context, various scientists and institutions have pioneered the development and application of organ-on-a-chip, each contributing to the growing body of knowledge in this field [10,18,[21], [22], [23]]. There has been a rapid rise in research publications related to this technology, leading to an enormous amount of information available [24]. This could be challenging for researchers new to this field or those looking to understand the overall trends and significant contributions in organ-on-a-chip technology. While traditional literature reviews and meta-analyses offer valuable perspectives, they frequently fall short in furnishing an exhaustive and integrated overview of a swiftly advancing domain.

Bibliometric analysis provides a solution to this challenge by using quantitative and qualitative methods to analyze scholarly publications [25,26]. It can identify leading researchers and institutions, highlight hot topics, chart the evolution of the field, and point towards future research frontiers. In recent years, bibliometric analysis has become increasingly popular in biomedical research due to the rise in scientific data and the availability of tools for such analyses.

Several bibliometric analyses have been performed in related fields such as organoids [27], 3D bioprinting [28], and tissue engineering [29]. Nevertheless, as far as we know, there is a dearth of bibliometric studies that specifically target organ-on-a-chip technology.

Therefore, in this study, we strive to bridge this informational void. We use several software tools and web platforms to analyze the literature on organ-on-a-chip and construct scientific knowledge maps. Our objectives are to (i) Determine the key figures in this field, encompassing countries, academic institutions, authors; (ii) track the evolution and development of research focus in the organ-on-a-chip field from 2014 to 2023; (iii) Anticipate the forthcoming research interests in this domain; (iv) provide new perspectives and ideas for future research on organ-on-a-chip; and (v) draw more attention to this emerging area of research. We believe this study could provide a valuable reference for clinicians and researchers engaged in this important and rapidly evolving field.

## 2 Methods

### 2.1 Data extraction

Data were obtained from the Web of Science Core Collection (WoSCC) database, a widely acknowledged citation-based repository extensively utilized in bibliometric research [[30], [31], [32]]. The data retrieval took place on July 18, 2023, encompassing the time span from January 1, 2014, to the date of the online search. Strict inclusion criteria were applied, limiting the selection to English-language papers with a focus on articles and reviews, while excluding alternative publication formats such as letters, meetings, abstracts. The search strategy was devised to encompass topics relevant to "organ-on-a-chip" and its synonyms (Supplement Fig. 1) [33]. The initial search yielded 3266 results, which were then subjected to a manual screening process. Two researchers (N.T. and C.R.H.) carefully examined the titles, abstracts, and full texts of the retrieved documents, excluding publications unrelated to the field of organ-on-a-chip. Consequently, 2305 eligible papers were determined and considered for subsequent study. The selection process is visually presented in Supplement Fig. 1.

### 2.2 Data analysis and visualization

Various software tools are commonly employed in bibliometric analyses, including VOSviewer, CiteSpace, and HistCite [34]. In this study, we utilized the most up-to-date versions of bibliometric tools, specifically VOSviewer 1.6.19, CiteSpace 6.1.R6, Biblioshiny 4.1.2, and an online site (https://bibliometric.com/), to ensure comprehensive and robust data analysis results. Prior to conducting the analysis, several data preprocessing steps were implemented. These steps encompassed the consolidation of synonyms into unified terms, the elimination of irrelevant terms, and the standardization of author and institution names that referred to the same entity but were spelled differently. By standardizing and optimizing the dataset, we aimed to enhance the accuracy and reliability of the subsequent analysis.

For bibliometric and visual analysis, we employed CiteSpace, a widely used tool in bibliometrics [35]. CiteSpace offers a suite of functionalities that facilitate comprehensive exploration and extraction of valuable insights from extensive academic literature. It enables researchers to visualize and analyze various aspects of scholarly research, including the identification of influential authors, institutions, and collaborations, the detection of emerging trends and research hotspots, and the tracking of citation patterns and co-citation networks. In our study, we leveraged CiteSpace for visualizing collaborations of inter-country and inter-institution, creating a dual-map overlay of journals, and conducting keyword cluster analysis. Additionally, CiteSpace were employed to identify the 30 most influential references with the strongest citation bursts. To ensure optimal analysis results, we adjusted the configuration options of CiteSpace in the subsequent manner: selection criteria (Top N = 50), pruning method (Minimum Spanning Tree and Pruning Sliced Networks), time span (2014–2023), and years per slice (n = 1), while the rest adhered to the standard settings. Centrality refers to the number of links going into or coming out of a node, a country or institution with a high degree centrality would be one that collaborates frequently with many other countries or institutions.

VOSviewer, a widely utilized software in bibliometric research [25], is employed to create co-occurrence maps. In our study, co-authorship, co-citation and co-occurrence maps were created by VOSviewer. In these maps, nodes or words of larger size indicate a higher frequency of occurrence, and the connection between nodes is represented by colored lines, with thicker lines denoting more extensive collaboration. Node colors represent variations in the average years of publication. To visualize the relationships among institutions, journals, and authors, we built a co-occurrence network diagram utilizing this tool. Additionally, clustering network maps were created based on the contributions of authors’ keyword, employing text data to gain valuable insights from the analysis.

For analyzing the literature related to the institution of attribution and authors, we utilized Biblioshiny version R4.1.2. This software facilitated a comprehensive examination of the relevant literature. Moreover, to create inter-country collaboration diagrams, we used the web tool available at https://bibliometric.com/. To create charts and conduct descriptive analysis on the annual publications, we employed Prism 9 (GraphPad, USA) and Excel (Microsoft Office, USA), respectively. Additionally, to evaluate the impact of the publications, we extracted the Impact Factor data from the 2022 Journal Citation Reports.

## 3 Results

### 3.1 Publication outputs and trends

**Fig. 1:**
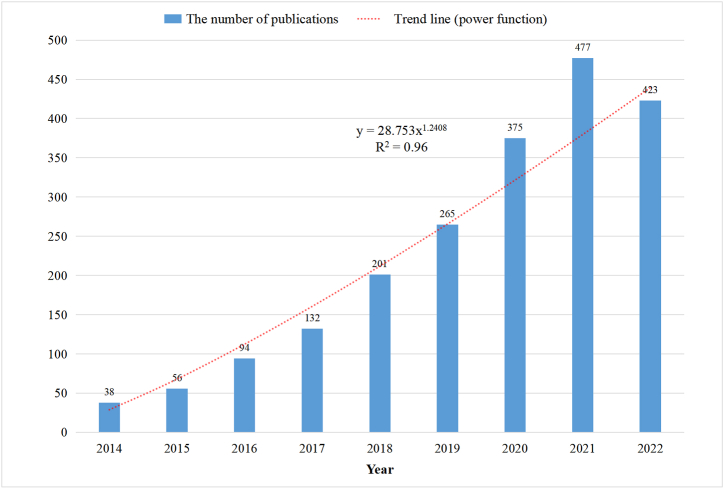
The corresponding number of annual publications regarding organ-on-a-chip from 2014 to 2023.

Using the screening strategy, we identified a total of 2305 eligible manuscripts. The yearly publication count for organ-on-a-chip research from 2014 to 2023 is depicted in Fig. 1. Notably, annual publication count demonstrated a significant growth pattern, crossing a landmark of 100 publications in 2017. To assess the changing trend more precisely, we developed a power function (y = 28.753x^1.2408, R^2^ = 0.96), X stands for the specific year, while Y denotes the number of publications for that year. This function provides a robust representation of the increasing publication rate over the study period.

### 3.2 Leading countries/regions and major funding bodies

**Fig. 2:**
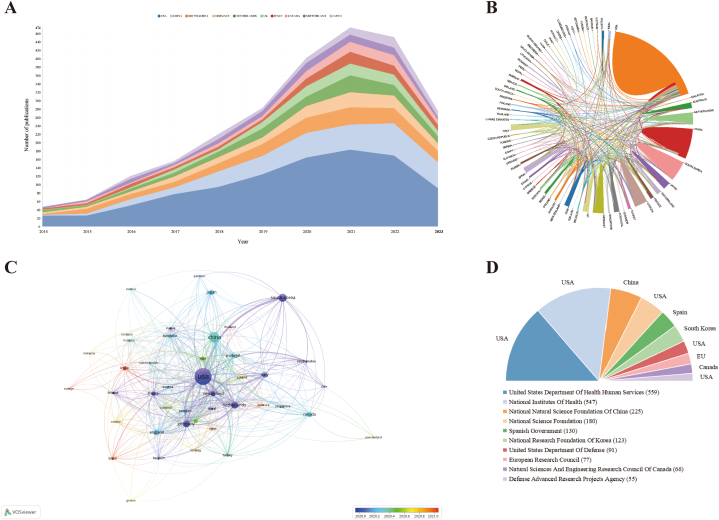
The distribution of counties/regions and funding agencies in organ-on-a-chip research. (A) The number of publications per year of the top 10 countries. (B) The international cooperation analysis. the line between two counties indicates cooperative relationship and thickness of the line represents the degree of collaboration (C) The overlay visualization of co-authorship. The size of notes/words represent the total publications of a country. The colour line between two nodes represents the degree of co-authorship. The colour of the nodes indicated different average publication year. (D) The top ten frequent funding agencies and corresponding countries.

A comprehensive analysis of publication data revealed 2249 distinct institutions spanning 75 nations/territories contributing to the publications. Table 1 showcases the leading ten countries and institutions in organ-on-a-chip researches. To analyze the yearly publication trends of these countries, Fig. 2A depicts the annual papers counts from 2014 to 2023. The United States (USA) holds the top position with a total of 1004 publications, subsequently by China (n = 358), South Korea (n = 225), Germany (n = 171), and the Netherlands (n = 162). USA stands out with the maximum centrality score of 0.24 (Table 1), indicating a strong influence and collaboration in this realm compared to other countries. In Fig. 2B, which delves into international collaboration, connections between countries denote partnerships in organ-on-a-chip investigations. USA shows dominant collaboration across nations within this field. The overlay visualization map (Fig. 2C) showcases nations or regions that have at least five publications, offering insights into their research networks and connections. Fig. 2D highlights the leading ten financial agencies, half of which originate from the USA. The United States Department of Health Human Services emerges as the most frequent funding source, providing support for a total of 559 publications in organ-on-a-chip research.

**Table 1:** The top 10 countries and institutions in organ-on-a-chip.

Rank	Country	Count	Centrality	Institution	Count	Centrality
1	USA	1004	0.24	Harvard Univ (USA)	213	0.14
2	China	358	0.01	MIT (USA)	65	0.14
3	South Korea	225	0.07	Univ Toronto (Canada)	59	0.09
4	Germany	171	0.19	Seoul Natl Univ (South Korea)	53	0.05
5	Netherlands	162	0.12	Chinese Acad Sci (China)	46	0.06
6	Italy	113	0.06	Southeast Univ (China)	44	0.02
7	England	110	0.13	Univ Twente (Netherlands)	39	0.09
8	Canada	109	0.03	Univ Washington (USA)	39	0.04
9	Switzerland	102	0.08	Boston Childrens Hosp (USA)	37	0.05
10	Japan	101	0.04	Univ Pittsburgh (USA)	36	0.04

### 3.3 Most productive institutions

**Fig. 3:**
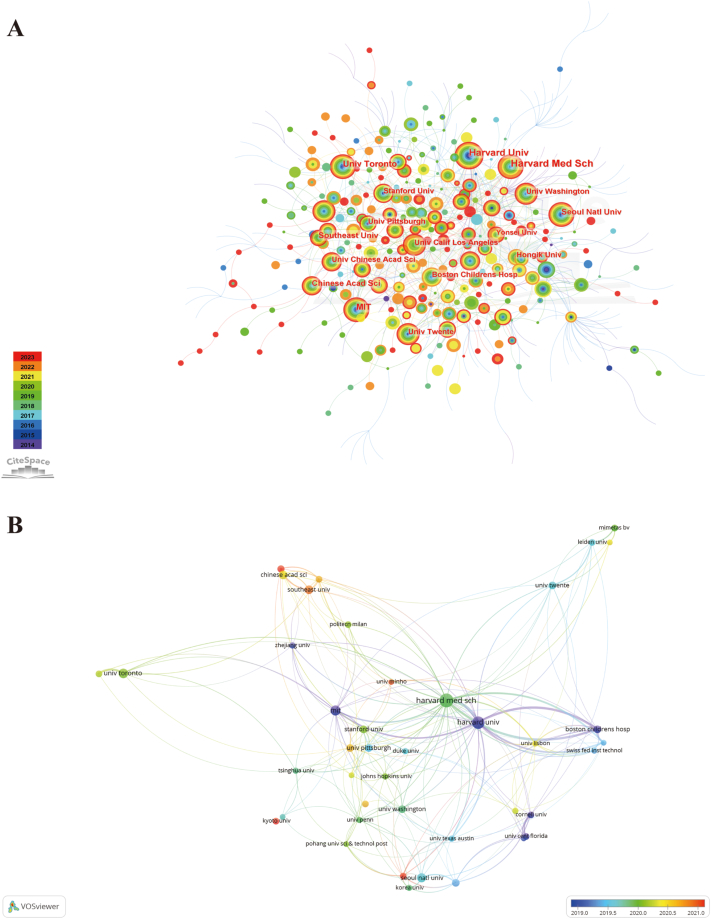
The distribution of institutions in organ-on-a-chip research. (A) Co-occurrences of institutions. The notes represent corresponding institution. The size of notes/words represent the total outputs of one institution. The value between lines indicates the cooperation degree among two connected institutions. (B) Overlay visualization map of co-authorship. The size of notes/words represent the total publications of an institution. The colour line between two nodes represents the degree of co-authorship. The colour of the nodes indicated different average publication year.

Fig. 3A illustrates the co-occurrences of institutions in organ-on-a-chip research, Table 1 displays the ten most productive institutions. Leading the list, Harvard University has the highest number of publications (n = 213), followed by Massachusetts Institute of Technology (n = 65), University of Toronto (n = 59), Seoul National University (n = 53), and Chinese Academy of Sciences (n = 46). Harvard University also stands out with the highest centrality score of 0.14, indicating its prominent role and collaborations regarding this area. Of the leading ten institutions, half are located in the USA. Furthermore, Fig. 3B presents an analysis of co-authorship among institutions. The color gradient in the analysis signifies the newer average publication year of institutions, with the color red assigned to the most recent publications.

### 3.4 Analysis of influential authors

**Fig. 4:**
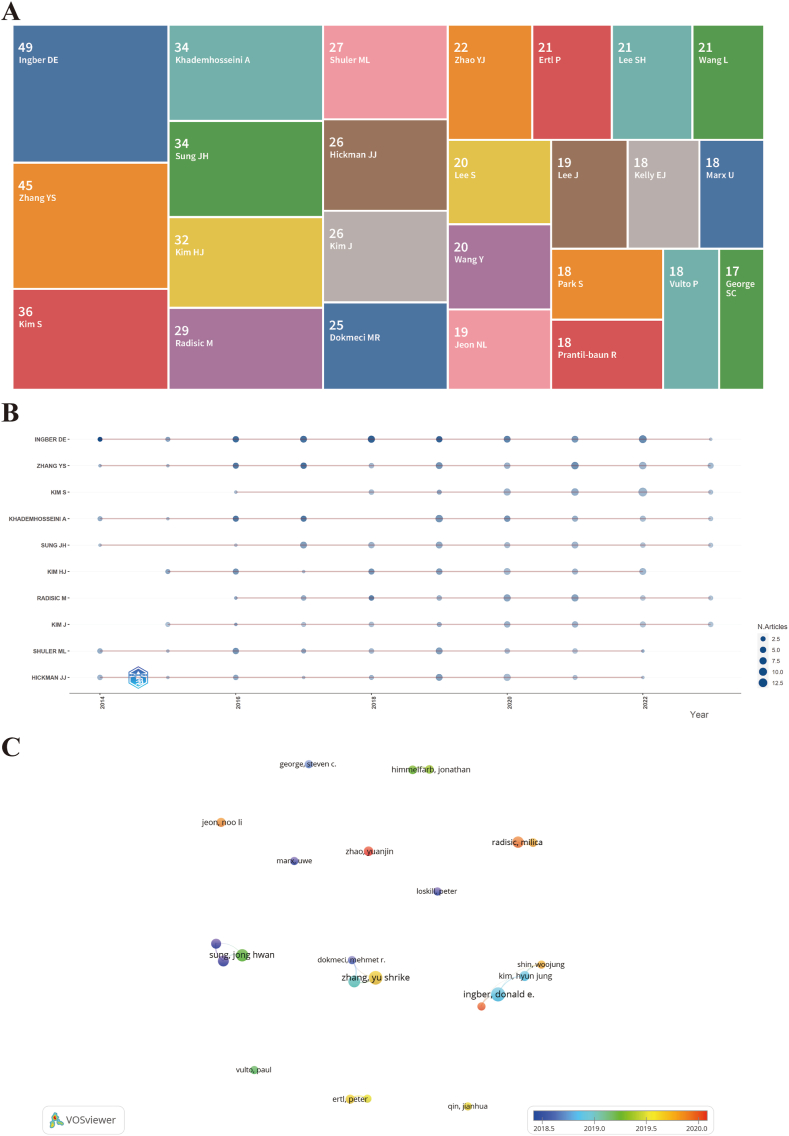
The overlay visualization of authors in organ-on-a-chip research. (A) Tree map of the top 25 productive authors. The number above the name represent the amount of the publications. (B) Top 10 authors' outputs over time. The size of the deep blue circle indicates the number of papers (N. papers). (C) The visualization of the top 20 prolific author relationships. The size of notes/words represent the total publications of an author. The colour line between two nodes represents the degree of co-authorship. The colour of the nodes indicated different average publication year.

Fig. 4A presents the list of the 25 leading authors in terms of article quantity in the organ-on-a-chip research field. Among these authors, the details of the top ten most prolific ones are visualized in Fig. 4B and summarized in Table 2. Fig. 4B provides a graphical representation of the yearly publication output for each of these authors. Ingber DE emerges as the most prolific author, boasting an impressive H-index valued at 33 coupled with an average citation count of 162.5319 per paper. However, it is noteworthy that despite having a smaller number of publications, Huh D remains the most co-cited author, indicating the significant impact of their work in the scholarly community. The relationships among the top 23 prolific authors are visualized in Fig. 4C, where we observe notable collaborations between Ingber DE, Kim HJ, and Shin W. The analysis of the most prolific authors in organ-on-a-chip research sheds light on their individual research productivity, influence, and collaborative partnerships within the scientific community.

**Table 2:** The top 10 most productive authors of organ-on-a-chip.

Rank	Authors	Count	Average citations per papers	H-index	Co-cited authors	Co-citations
1	Ingber DE	49	162.5319	33	Huh D	1442
2	Zhang YS	45	68.4545	27	Kim HJ	766
3	Khademhosseini A	34	87.9394	24	Sung JH	591
4	Sung JH	34	33.3235	18	Bhatia SN	561
8	Kim HJ	32	84.6087	19	Zhang YS	536
5	Radisic M	29	65.5862	18	Jang KJ	434
6	Shuler ML	27	82.0741	19	Skardal A	418
7	Hickman JJ	26	51.1667	17	Zhang BY	402
9	Zhao YJ	22	59.2727	15	Esch MB	391
10	Ertl P	21	36.0526	13	Benam KH	373

**Fig. 5:**
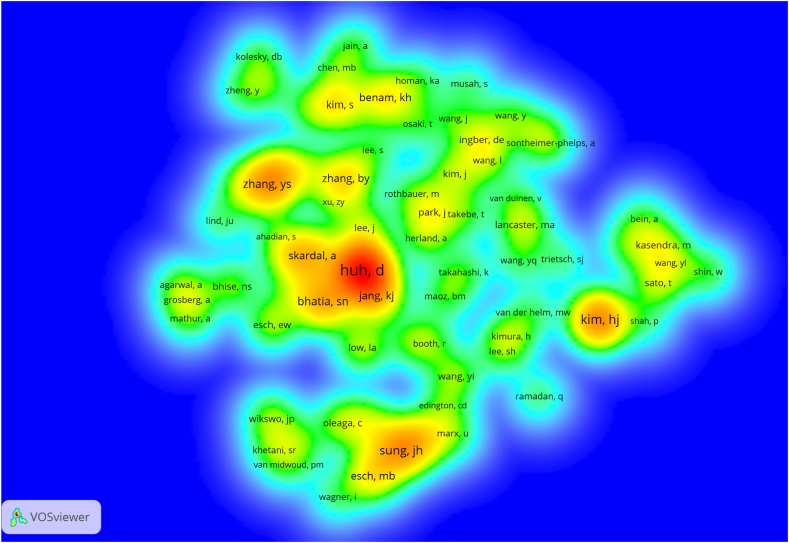
The density visualization of co-cited authors in organ-on-a-chip research. The size of the circle/word are positively related to the number of co-citations.

Fig. 5 displays the density visualization of authors through co-citation patterns, with a focus on authors who have received ≥120 co-citations. Among the authors with the highest co-citations, Huh D leads the list with an impressive count of 1442 co-citations. Following closely are Kim HJ with 766 co-citations and Sung JH with 591 co-citations. The complete roster of the leading ten authors in co-citation, along with their respective co-citation counts, can be found in Table 2. These authors have received significant recognition and acknowledgment within the research community, as evidenced by the substantial number of co-citations they have garnered. The analysis of most prolific and co-cited authors sheds light on their individual research productivity, influence, and collaborative partnerships within the scientific community.

### 3.5 Most active journals

**Fig. 6:**
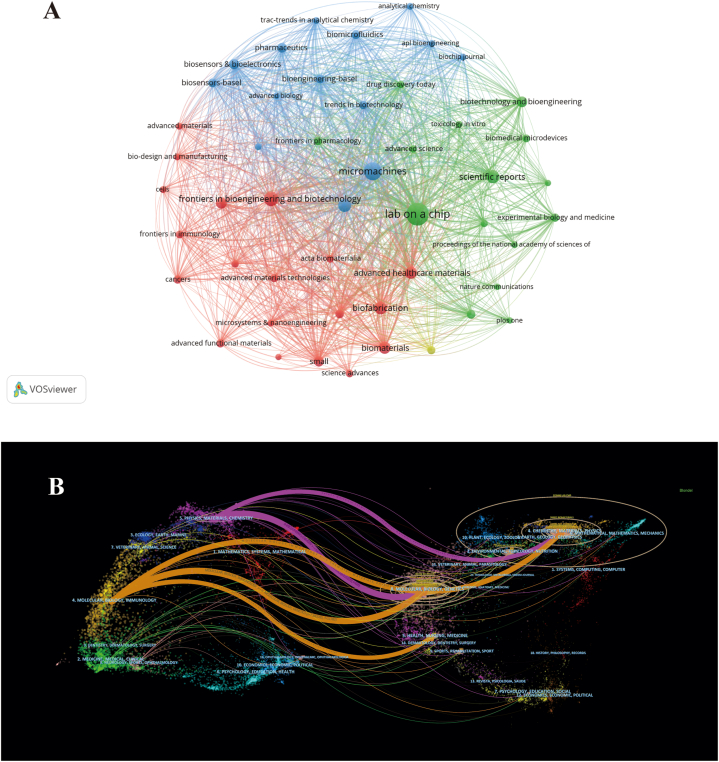
The visualization of journals in organ-on-a-chip research. (A) Network visualization map of journals analysis. The size of notes/words represent the total publications of a journal. The colour line between two nodes represents the degree of co-citation. The colour of the nodes indicated different average publication year. (B) Dual-map overlay of journals. The citing journals are on the left, the cited journals are on the right. The colored path represents citation association of journals.

The analysis covered a total of 612 journals that featured articles relevant to the research. Among these, 49 journals were identified to have published at least 10 papers and were included in the visualization, as shown in Fig. 6A. Table 3 presents the ten productive journals along with their essential details. The journal with the highest publication count is "Lab on a Chip" (n = 158), followed by "Micromachines" (n = 93), and "Frontiers in Bioengineering and Biotechnology" (n = 82). Notably, every journal within the top ten boasted an Impact Factor (IF) for the year 2022 greater than 3. Additionally, eight of these journals were classified within the Q1 category according to the Journal Citation Reports (JCR) division. For a comprehensive visualization of journal connections, a dual-map overlay was generated using CiteSpace, as depicted in Fig. 6B. This visualization identified five citation paths, offering insights into the interconnections and relationships among the included journals.

**Table 3:** The top 10 most productive journals in organ-on-a-chip.

Rank	Journal	Count	IF 2022	JCR quartile 2022
1	LAB ON A CHIP	158	6.1	Q1
2	MICROMACHINES	93	3.4	Q2
3	FRONTIERS IN BIOENGINEERING AND BIOTECHNOLOGY	59	5.7	Q1
4	ACS BIOMATERIALS SCIENCE AND ENGINEERING	47	5.8	Q2
5	SCIENTIFIC REPORTS	46	4.6	Q1
6	BIOFABRICATION	45	9.0	Q1
7	ADVANCED HEALTHCARE MATERIALS	41	10.0	Q1
8	BIOMATERIALS	39	14.0	Q1
9	INTERNATIONAL JOURNAL OF MOLECULAR SCIENCES	33	5.6	Q1
10	BIOTECHNOLOGY AND BIOENGINEERING	30	3.8	Q1

### 3.6 Top cited articles and Co-cited references and reference burst

**Fig. 7:**
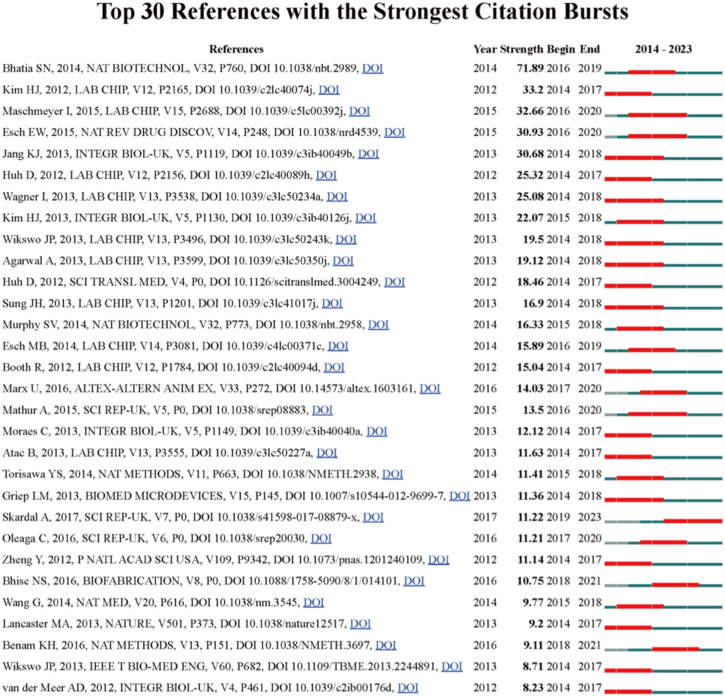
Top 30 references with the strongest citation bursts in organ-on-a-chip research. Ranked by strength. The bars in red stands for a burst period for the references

In the supplementary material, we provided a list of the top 100 most cited articles in Supplement Table 1. Among these, the ten publications with the highest citation counts have garnered more than 450 citations each. Furthermore, Table 4 presents a summary of the top 10 most co-cited references. Among these papers, two references received co-citations exceeding 500 instances. Additionally, we performed an analysis of citation bursts with CiteSpace to pinpoint papers experiencing significant spikes in citations over the past ten years (Fig. 7). Remarkably, the article authored by Bhatia, SN in 2014 emerged as the top cited article and ranked as one of the most co-cited references in the organ-on-a-chip field, exhibiting the highest citation strength (strength = 71.89). This article received significant attention, primarily between 2016 and 2019 [1].

**Table 4:** The Top 10 Most Co-cited References in organ-on-a-chip.

Rank	Title (publication year)	First author	Journal	Co-citations
1	Reconstituting organ-level lung functions on a chip (2010)	Dongeun Huh	Science	614
2	Microfluidic organs-on-chips (2014)	Sangeeta N Bhatia	Nature biotechnology	524
3	Human gut-on-a-chip inhabited by microbial flora that experiences intestinal peristalsis-like motions and flow (2012)	Hyun Jung Kim	Lab on a chip	332
4	From 3D cell culture to organs-on-chips (2011)	Dongeun Huh	Trends in cell biology	248
5	Organs-on-chips at the frontiers of drug discovery (2015)	Eric W Esch	Nature review	233
6	A four-organ-chip for interconnected long-term co-culture of human intestine, liver, skin and kidney equivalents (2015)	Ilka Maschmeyer	Lab on a chip	230
7	A human disease model of drug toxicity-induced pulmonary edema in a lung-on-a-chip microdevice (2012)	Dongeun Huh	Science translational medicine	226
8	Contributions of microbiome and mechanical deformation to intestinal bacterial overgrowth and inflammation in a human gut-on-a-chip (2016)	Hyun Jung Kim	Proceedings of the National Academy of Sciences of the United States of America	219
9	Human kidney proximal tubule-on-a-chip for drug transport and nephrotoxicity assessment (2013)	Kyung-Jin Jang	Integrative biology	210
10	Small airway-on-a-chip enables analysis of human lung inflammation and drug responses *in vitro* (2016)	Kambez H Benam	Nature methods	193

### 3.7 Analysis of co-occurring author keywords

**Fig. 8:**
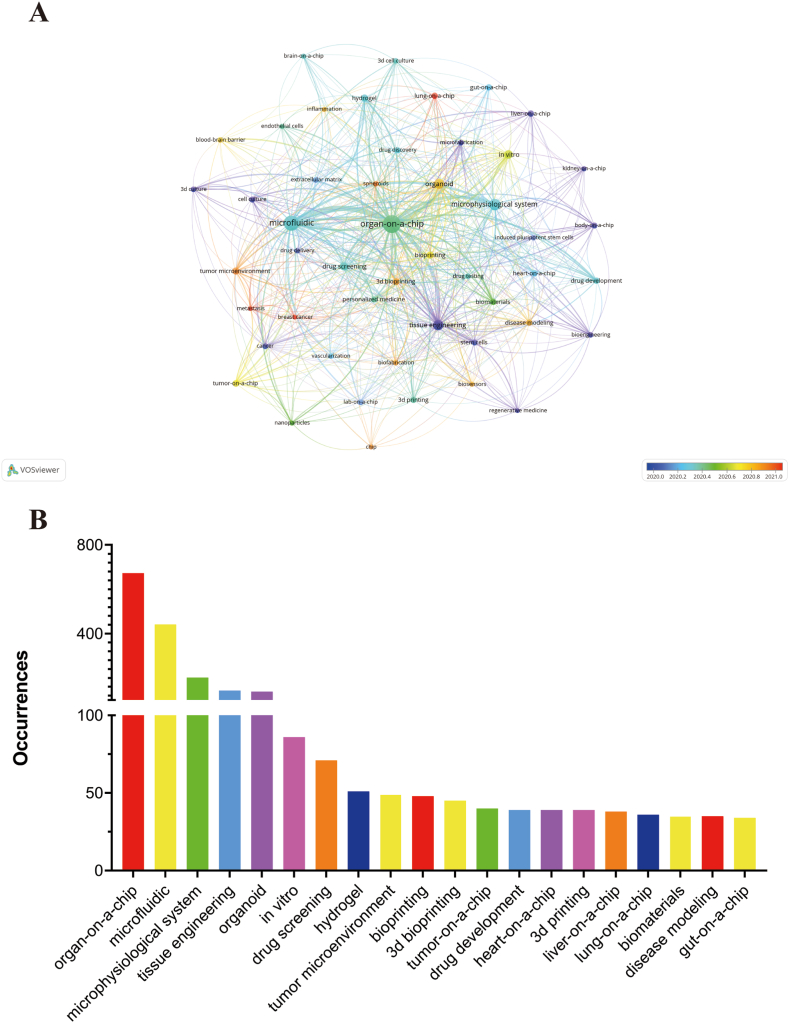
(A) The overlay of author keywords in organ-on-a-chip research. The size of notes/words represent occurrences. The colour of the nodes indicated average publication year based on the strip of colour. (B) Top 20 author keywords with most frequent occurrences in organ-on-a-chip research.

Keywords serve as essential indicators of the research direction and focus areas, enabling researchers to stay abreast of the latest developments and advancements in this rapidly evolving field [36,37]. In our study, we employed author keywords to achieve a thorough insight into the dominant research topics and emerging trends within the domainof organ-on-a-chip. Out of the 4443 author keywords identified, we concentrated on the 49 keywords that appeared at least 18 times, resulting in the overlay visualization map shown in Fig. 8A. The visualization map highlights the interconnections and relationships among these selected keywords, revealing the key areas of interest and research focus within the domain of organ-on-a-chip. Moreover, Fig. 8B displays the top 20 author keywords based on their frequency of occurrence. Notably, keywords such as “Organ-on-a-chip”, “microfluidic”, “microphysiological system”, “tissue engineering”, “organoid”, “*in vitro*”, “drug screening”, “hydrogel”, “tumor microenvironment”, “bioprinting” emerged as the most prominent and frequently used terms.

**Fig. 9:**
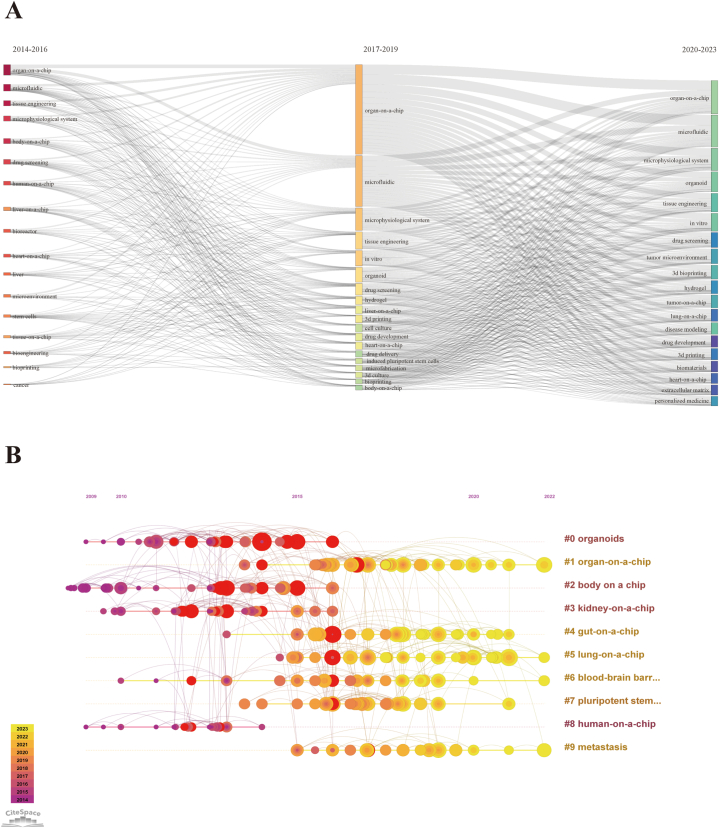
(A) Three-field plot of author keywords in organ-on-a-chip research. The thick of the notes represent the relative co-occurrence frequency in a specific period. (B) The timeline view of co-cited reference in organ-on-a-chip research. Years from 2009 to 2022 are arranged horizontally at the top, 10 clusters based on author keyword were identified and listed on the right. The larger the size of the circle, the more studies on the theme.

Furthermore, Fig. 9A depicts the thematic evolution analysis. We identified the top 19 author keywords with the highest occurrences in their respective periods. To illustrate the evolution of the organ-on-a-chip field over three distinct time periods, a three-field plot was utilized. Of particular significance, our analysis highlights that the keywords "tumor microenvironment" and "lung-on-a-chip" have received substantial attention in the last two years. These topics have emerged as focal points of interest and research within the organ-on-a-chip domain, indicating their growing importance and relevance in recent studies.

Fig. 9B illustrates nodes that denote co-cited references, plotted on a timeline-view graph.Through an analysis of the author keywords, the co-cited references were categorized into ten distinct clusters with specific themes (Modularity Q value = 0.62, Weighted mean silhouette value = 0.84). These clusters include: "organoids" (#0), "organ-on-a-chip" (#1), "body on a chip" (#2), "kidney-on-a-chip" (#3), "gut-on-a-chip" (#4), "lung-on-a-chip" (#5), "blood-brain barrier" (#6), "pluripotent stem cell" (#7), "human-on-a-chip" (#8), and "metastasis" (#9). Remarkably, "metastasis" and "lung-on-a-chip" represent two emerging research directions within the field of organ-on-a-chip in recent years. These novel areas of investigation signify the evolving research landscape, reflecting the scientific community's growing interest in understanding and simulating metastasis and exploring lung-on-a-chip models for various applications.

**Fig. 10:**
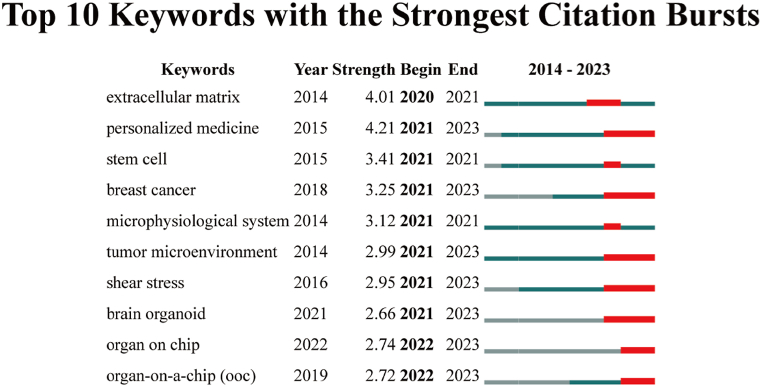
Top 20 author keywords with the strongest citation bursts in organ-on-a-chip research. Ranked by begin year of citation burst. The bars in red stands for a burst period for the keywords.

Additionally, Fig. 10 displays the top 10 author keywords with the most significant citation bursts. Among these, "breast cancer," "tumor microenvironment," "shear stress," and "brain organoid" are four keywords that have experienced citation bursts in the recent 2 years. These keywords are indicative of the prevailing research trends in this domain, reflecting the areas that have gained increased attention and interest in recent times. The clustering method used in the analysis exhibited a modularity Q of 0.62 and a weighted mean silhouette value of 0.84, validating the rationality and effectiveness of this approach in grouping and identifying meaningful clusters based on the citation patterns.

## 4 Discussion

### 4.1 Overview and key insights

The yearly publications growth and the quantity of citations are essential metrics for identifying research trends in scientific fields [38]. By scrutinizing the global distribution of scientific publications, we attain a more profound comprehension of the attributes and present state of global scientific contributions [39]. This study spotlights the burgeoning research fronts and the evolving research foci in the organ-on-a-chip domain over the preceding decade. The data, mined from the WOSCC database, manifests that 10,576 authors affiliated with 2249 institutions across 75 countries/regions, published 2305 papers pertaining to organ-on-a-chip in 612 distinct journals from January 1, 2014 to July 18, 2023.

From the trend-line depicted in Fig. 1, there's a notable increase in the number of journal articles. Prior to 2014, the annual publication count did not exceed 40, which can be ascribed to the then embryonic status of the organ-on-a-chip paradigm and the persisting technical challenges that were yet to be surmounted [1]. However, with the evolution and commercialization of microfluidic devices and in-vitro systems, the organ-on-a-chip field experienced a surge in research, thereby accounting for the subsequent exponential growth in related studies post-2014 [1,4,40,41]. Extrapolating from this growth trajectory, we conclude that the organ-on-a-chip technology has elicited substantial research interest and attention in recent years, and we anticipate a continued escalation in the volume of papers within this field.

### 4.2 Geographical and institutional contributions

Our analyses of the geographic distribution and institutional contributions, as depicted in Fig. 2, highlight USA and China as the most prolific contributors. This underlines the United States' significant contributions and leading position in the organ-on-a-chip research domain. Beyond the organ-on-a-chip research subject, previous bibliometric papers have revealed the USA' predominant publishing output in other realms of organoid or tissue engineering research [42,43]. It is well-documented that research output positively correlates with the gross domestic product (GDP) [44,45]. Furthermore, it is noteworthy that China and the USA allocate the most significant financial support for research in this domain, resulting in a 1.2-fold more publications than the combined all of the next top 10 countries South Korea, with a publication count exceeding 200, maintains close scholarly exchange with the United States, underscoring the importance of international collaboration in advancing this field.

Analogous to the geographic distribution, the leading ten institutions with the highest publication output are primarily located in the USA and China. Renowned research institutions such as Harvard University, MIT, the Chinese Academy of Sciences, Southeast University, the University of Washington, Boston Children's Hospital, and the University of Pittsburgh, occupy seven of the top ten slots in the list of most prolific institutions. It is apparent that leading academic institutions often spearhead advancements in most fields. Harvard University's centrality score of 0.14 indicates its pivotal role in the organ-on-a-chip research landscape. Furthermore, the active international cooperation between various institutions underscores the significance of collaboration in fostering growth within this field [46].

### 4.3 Leading scholars and citation networks

Identifying influential scholars in a particular field provides early-career researchers with insightful guidance and direction [47]. In the data presented in Fig. 4 and enumerated in Table 2, we highlight the authors with the highest number of publications. Dr. Donald E. Ingber from the Wyss Institute for Biologically Inspired Engineering at Harvard University leads the list with 49 articles and an average citation count of 162.53 per paper. As the Founding Director of the Wyss Institute, Dr. Ingber and his team have pioneered multiple innovative technologies, including organ-on-a-chip and programmable nanomaterials, achieving considerable progress in areas such as lung-on-a-chip and gut-on-a-chip systems [4,41,48]. As a globally recognized expert in organ-on-a-chip technology, Dr. Ingber has created several significant achievementsin this specialty, including the development of lung-on-a-chip, a device that mimics the mechanical and biochemical behaviors of a human lung [4].

Another researcher of particular significance is Dr. Yu Shrike Zhang from the Department of Medicine at Harvard Medical School and Brigham and Women's Hospital, who has published 45 articles with an average citation count of 68.45 per paper and an H-index of 27. Dr. Zhang's seminal contributions to the organ-on-a-chip field include the development of 3D bioprinted *in vitro* models of human tissues using microfluidic systems [49], and the advancement of biomimetic liver-on-chip platforms [8]. There is no doubt that Dr. Zhang's work has greatly advanced the integration of bioengineering and personalized medicine.

Importantly, all top ten authors in the field have had notable achievements each year, suggesting a vibrant future for the organ-on-a-chip field. Contrary to expectations, the degree of collaboration among the 25 leading authors in productivity appears limited, perhaps due to differences in geographic location and institutional affiliation, or due to impacts such as the COVID-19 pandemic.

With respect to co-cited authors, as depicted in Fig. 5 and Table 2, Dr. Dongeun Huh from the Department of Bioengineering at the University of Pennsylvania and Dr. Hyun Jung Kim from the Department of Chemical Engineering at the University of Texas at Austin have both authored several highly cited review papers on organ-on-a-chip technologies [50,51]. These comprehensive review articles serve as invaluable references, aiding other researchers in rapidly and accurately understanding the dynamically evolving field of organ-on-a-chip. In essence, the top 10 authors, based on their influential publications and co-citations, are driving advancements in the field.

### 4.4 Journals of influence

Journals serve as a significant conduit for the diffusion of academic research findings [52]. In our research, we have encapsulated the visualization network of co-citations representing the leading journals in the organ-on-a-chip arena. This can guide researchers in choosing the most appropriate journals for their manuscript submissions. As outlined in Fig. 6 and Table 3, Lab on a Chip, Micromachines, Frontiers in Bioengineering and Biotechnology, Biotechnology, ACS Biomaterials Science and Engineering, Scientific Reports, Biofabrication, Advanced Healthcare Materials, Biomaterials, International Journal of Molecular Sciences and Biotechnology and Bioengineering occupy the top positions. Lab on a Chip has published the most papers on organ-on-a-chip research, and is a leading journal in the field that mainly focuses on the development and application of miniaturized systems. At the same time, journals from the JCR Q1 category constitute 80 % of the top 10 journals. This indicates that these publications attract significant attention from researchers and are central to the field of organ-on-a-chip research.

The dual-map overlay depicts the thematic spread across academic journals. Fig. 6 delineates 5 citation paths, Molecular/Biology/Immunology co-cited journals to Chemistry/Materials/Physics, Molecular/Biology/Genetics and Health/Nursing/Medicine; and Physics/Materials/Chemistry co-cited journals to Molecular/Biology/Genetics and Chemistry/Materials/Physics. This represents the thematic distribution of the associated journals. The citing journals are positioned on the left, and the cited journals are on the right. The saffron and purple paths reflect the relationships between the citing and cited journals, respectively. This outcome indicates that organ-on-a-chip is a critical interdisciplinary field, and the current mainstream research direction is the collaboration between Chemistry/Materials/Physics and Molecular/Biology/Genetics/Immunology.

### 4.5 Top ten seminal publications

A co-citation reference suggests that a particular publication has been cited by different papers [53]. The fundamental knowledge for this study includes references acknowledged by the papers considered in the analysis, which isn't necessarily identical to a highly cited paper.

In light of the references outlined in Table 4, 20 % of the papers are review articles. The journal Science published the most co-cited paper, "Reconstituting organ-level lung functions on a chip," by Dr. Dongeun Huh in 2010, which has garnered a total of 614 co-citations to date [4]. This landmark work made a significant contribution to the field of organ-on-a-chip research by demonstrating the potential of chip-based systems to replicate complex organ-level functions. This study focused on reconstituting the physiological and mechanical functions of a human lung on a microchip. This influential piece has undoubtedly been instrumental in paving the way for the comprehensive development of the organ-on-a-chip field.

Four years subsequent to the publication of "Reconstituting organ-level lung functions on a chip", the second most co-cited paper in the organ-on-a-chip field was published by Dr. Donald E. Ingber and his team [1]. The team presented a seminal review titled "Microfluidic organs-on-chips" in Nature Biotechnology. In this extensive review, they broadened the concept of organ-on-a-chip technology and discussed various microfluidic organ-on-chip models. This work underscored the potential of these microdevices to emulate the physiological responses of multiple human organs. The team also highlighted advancements in the field, including the development of lung, gut, liver, and kidney chips. By summarizing state-of-the-art progress in the organ-on-a-chip field and outlining potential future developments, this paper served as an important guide for subsequent research and commercial applications in the field.

The third most co-cited paper in the field of organ-on-a-chip research is "Human gut-on-a-chip inhabited by microbial flora that experiences intestinal peristalsis-like motions and flow", authored by Hyun Jung Kim, and published in 2012 in Lab on a Chip [41]. This paper introduced a novel gut-on-a-chip that was inhabited by living microbes and was able to recreate peristaltic motions and fluid flow, mimicking the physical microenvironment of the human gut. This work highlighted the potential of organ-on-a-chip technology to advance beyond traditional cell culture and animal models, offering a more physiologically relevant platform for drug testing and disease modeling. The other highly co-cited articles among the top 10 were published from 2011 to 2016, as listed in Table 4.

### 4.6 References exhibiting notable citation bursts

The references with high burst values in a specific timeframe suggest a surge in attention during that period. The top 30 references with the strongest citation bursts in the organ-on-a-chip field are listed in Fig. 7.

Unsurprisingly, the reference with the most substantial citation burst was the pivotal paper "Microfluidic organs-on-chips" by Dr. Sangeeta N Bhatia, published in 2014 in Nature Biotechnology, displaying a burst strength of 71.89 [1]. This reaffirms the significant influence of this groundbreaking work in the domain of organ-on-a-chip research.

Another highly cited burst reference was the innovative paper "Human gut-on-a-chip inhabited by microbial flora that experiences intestinal peristalsis-like motions and flow" by Hyun Jung Kim, published in Lab on a Chip in 2012 [41]. This paper exemplified the scientific prowess of organ-on-a-chip technology by replicating the complex functionalities of the human gut, further emphasizing its potential in the future of biomedical research, disease modeling, and drug testing.

The citation burst timeframe for these 30 references started after 2014 and ended approximately in 2023. This indicates that the organ-on-a-chip technology, as an innovative interdisciplinary field, has experienced rapid development over the past decade. Emerging as a hotspot in biomedical engineering, drug discovery, and personalized medicine, its potential to recapitulate human organ structure, functionality, and physiological responses *in vitro* has garnered global attention, ignited prolific scientific discussions, and inspired a wealth of innovative research endeavors [[54], [55], [56], [57]].

### 4.7 Current trends and future directions in organ-on-a-chip research

Analysis of author keyword occurrences sheds light on the predominant areas of interest and emerging trends in the organ-on-a-chip field. High occurrence author keywords, as illustrated in Fig. 8, include Organ-on-a-chip, Microfluidic, Microphysiological System, Tissue Engineering, Organoid, *In Vitro*, Drug Screening, Hydrogel, Tumor Microenvironment, Bioprinting, 3D Bioprinting, Tumor-on-a-Chip, Drug Development, Heart-on-a-Chip, 3D Printing, Liver-on-a-Chip, Lung-on-a-Chip, Biomaterials, Disease Modeling, Gut-on-a-Chip. These represent the research hotspots in the organ-on-a-chip landscape [[58], [59], [60]].

Moreover, Tumor-on-a-chip, Heart-on-a-chip, Liver-on-a-chip, and Lung-on-a-chip have received the highest average citations. Their popularity reflects the diverse applications of organ-on-a-chip models in studying various diseases and physiological conditions [10,[61], [62], [63]].

The shifts in research hotspots over time, depicted in Fig. 9A, allow us to discern future trends in the field. Comparing the research hotspots over the past ten years to those in the recent four years reveals significant changes. For instance, 3D bioprinting and organoids have drawn increasing attention, indicating the growing intersection of organ-on-a-chip technology with these areas [5,12,28,43,[64], [65], [66]]. In line with this trend, the importance of biomaterials, such as hydrogels, has also risen given their pivotal role in creating physiologically relevant *in vitro* models [22,[67], [68], [69], [70], [71], [72]].

Based on this bibliometric analysis, we foresee that Organ-on-a-chip, Microfluidics, 3D Bioprinting, Personalized Medicine, and Disease Modelling will remain at the forefront of research in the coming years. The field is likely to continue evolving towards the integration of more advanced technologies, the development of more complex and physiologically relevant models, and broader applications in disease modelling and personalized medicine.

#### 4.7.1 Exploration of metastasis in tumor microenvironments

Metastasis is a critical factor in cancer progression and accounts for most cancer-related deaths. The tumor microenvironment plays a crucial role in this process, with the interactions between cancer cells and their surroundings influencing metastatic behavior [73]. Understanding the underlying mechanisms of metastasis can, therefore, contribute to developing more effective cancer therapies.

Organ-on-a-chip technology facilitates the development of 3D tumor models that recapitulatefundamental elements of the tumor microenvironment, encompassing intricate interactions between cells and between cells and the extracellular matrix [74,75]. This is a significant improvement over conventional 2D cell culture models, which fail to mimic these interactions accurately. Furthermore, the dynamic nature of organ-on-a-chip models allows for the study of how fluid shear stress and interstitial flow, physiological parameters often overlooked in static 2D and 3D cultures, affect tumor progression and metastasis.

These chip-based systems also allow for co-culturing of various cell types, mimicking the heterogeneity of the tumor microenvironment more closely [76]. For instance, the co-culture of cancer cells with endothelial cells in a lung-on-a-chip can simulate the process of extravasation, an essential step in metastasis. Similarly, a liver-on-a-chip system can be utilized to study the final stage of metastasis, colonization, in which disseminated tumor cells adapt to and proliferate in the new organ [77].

Moreover, organ-on-a-chip technology enables the study of patient-specific tumor models, thereby facilitating personalized medicine approaches in cancer treatment [78]. By sourcing patient-derived cells for the chip-based systems, researchers can investigate the patient-specific mechanisms of metastasis and response to therapeutics. This capability may lead to the development of personalized treatment strategies aimed at preventing or limiting metastasis.

#### 4.7.2 Emergence of organoids

The integration of organ-on-a-chip technology and organoids is a promising research area with potential to significantly advance our understanding of human biology and disease, and fuel the development of novel therapeutics [14,63,79,80]. Organoids, being 3D tissue cultures derived from stem cells, can mimic the micro-anatomy of their tissue of origin. When integrated into organ-on-a-chip systems, these biomimetic microsystems simulate the functions and physiological responses of organs, thus enhancing the authenticity of these models [81].

Such integration provides an exciting opportunity for personalization. Organoids can be generated from patient-specific induced pluripotent stem cells (iPSCs), facilitating the creation of personalized organ-on-a-chip models for drug testing and disease modeling [10,82,83]. Moreover, organoids enhance disease modeling as they can replicate the microarchitecture and functionality of various organs, providing an organ-specific and patient-specific study approach [49].

However, technical challenges exist, including difficulties in integrating organoids into microfluidic devices, maintaining organoid cultures, and ensuring reproducibility and scalability. Despite these challenges, the union of organoids with organ-on-a-chip technology holds great potential for enriching our knowledge of human biology and accelerating therapeutic discoveries.

#### 4.7.3 Advances in tissue engineering

Organ-on-a-chip technology offers a profound advancement in the realm of tissue engineering by enabling the fabrication of multi-cellular systems that closely emulate human physiology [22]. This technology facilitates the precise recreation of human tissue microenvironments, significantly enhancing our capacity to model disease progression, evaluate therapeutic responses, and optimize tissue-engineered constructs [84].

Moreover, these microsystems allow a high degree of environmental control, including nutrient delivery, waste removal, and mechanical force application, fostering the engineering of tissues that more accurately reflect their in vivo counterparts [15]. Additionally, the co-culture of different cell types facilitates the understanding of tissue interactions, advancing the development of multi-tissue or organ system models [[84], [85], [86]].

However, the amalgamation of tissue engineering with organ-on-a-chip technology is not devoid of challenges. Standardization of these models, ensuring reproducibility, scalability, and the high associated costs represent some of the hurdles faced by researchers in the field.

Nonetheless, the integration of organ-on-a-chip technology with tissue engineering offers a promising avenue for the advancement of personalized therapies, improved drug discovery processes, and a deeper understanding of organ development and disease mechanisms.

#### 4.7.4 Developments in bioprinting and 3D bioprinting techniques

Bioprinting, with a specific focus on 3D bioprinting, is a crucial component in the progression of organ-on-a-chip technologies. This fabrication technique allows for the generation of sophisticated and intricate cellular structures, a critical requirement for accurately simulating human physiology and pathology [64]. 3D bioprinting's potential to create complex cellular and extracellular environments with high spatial resolution significantly advances the capabilities of organ-on-a-chip models [87]. This technology allows for the precise placement of diverse cell types in specified arrangements, replicating the unique cellular composition of human tissues and organs [47].

An integral challenge in tissue engineering and organ-on-a-chip technology is the creation of vascular networks necessary for supplying nutrients and oxygen to cells. Bioprinting can construct these complex vascular networks, enhancing the viability and functionality of engineered tissues within organ-on-a-chip systems [64,65]. The versatility of 3D bioprinting extends to customization, enabling the construction of patient-specific or disease-specific organ-on-a-chip systems. This capability improves disease modeling accuracy and potentially enhances drug testing platforms, aligning with personalized medicine goals [8].

Nevertheless, the need for biocompatible, biodegradable materials for cell support, improved resolution for better micro-scale replication, and faster printing speeds for large-scale applications represent ongoing hurdles. In spite of these challenges, the intersection of bioprinting and 3D bioprinting with organ-on-a-chip technology has already demonstrated tangible progress in the field. As advancements continue, these technologies promise to deliver increasingly accurate and practical organ-on-a-chip models, contributing to the broader realization of personalized medicine.

#### 4.7.5 The critical role of biomaterials in organ-on-a-chip technology

The development of organ-on-a-chip technology has been significantly bolstered by advancements in biomaterials. These materials have been integral in the creation of *in vitro* microenvironments that accurately mirror native tissue structure and function. Among these biomaterials, hydrogels are widely used due to their ability to simulate the extracellular matrix (ECM) of tissues [88,89]. By mimicking the ECM, hydrogels offer structural support and influence cell behavior, enhancing the realism of organ-on-a-chip models. Bioinks, another category of biomaterials used in 3D bioprinting, enable the generation of complex multicellular structures that resemble the architecture of specific organs [64,65]. This capacity has allowed for a higher degree of physiological relevance in organ-on-a-chip systems. However, improved understanding of tissue-specific matrix compositions and mechanical properties is needed for the development of more accurate biomaterials. The search for biomaterials that balance biocompatibility with adjustable mechanical, electrical, and biological properties is ongoing. In summary, biomaterials have been fundamental in enhancing organ-on-a-chip technology and hold great promise for the future of disease modeling, drug discovery, and personalized medicine.

#### 4.7.6 Personalized and precision medicine: a new frontier

Organ-on-a-chip technology has significantly bolstered the progress of personalized and precision medicine. Organ-on-a-chip technology holds immense potential for drug screening. By constructing different tissue organoids and disease models, the efficacy of new drugs can be explored in a patient-specific and disease-specific context. This is a burgeoning area of research with significant implications for the discovery and development of more effective and personalized therapeutic agents [90]. Moreover, the use of organ-on-a-chip technology to create personalized organ models stands to revolutionize transplant medicine [91]. With the capacity to build patient-specific organ chips, this technology could potentially reduce organ rejection rates and improve post-transplant recovery [29]. This approach aligns with the broader objectives of personalized and precision medicine and is likely to be a key area of focus in future research. The development of organ-on-a-chip technology offers exciting prospects for the advancement of personalized and precision medicine. Whether through enhancing drug screening processes, or paving the way for better outcomes in transplant, the potential applications of this technology are vast. Continued research and development in this field promise to yield even more innovative solutions to some of the most pressing challenges in healthcare today.

### 4.8 Strengths and limitations

Compared to earlier investigations that solely relied on meta-analysis or narrative reviews, this study boasts several advantages. Significantly, it represents the first bibliometric analysis mapping and characterizing the intellectual landscape of organ-on-a-chip research from 2014 to 2023. Various bibliometric software and instruments were utilized for this analysis, providing a multi-faceted and comprehensive view of the field. Moreover, rigorous quality checks were conducted on the literature included in the final analysis, ensuring the reliability of our results.

However, our work is not without limitations. Firstly, the data on organ-on-a-chip were primarily gathered from the WOSCC database, potentially missing out on some relevant publications not included in this database. Despite this, WOSCC is frequently selected as the go-to database for conducting bibliometric evaluations, and its data are generally accepted to be a representative sample of the most publications in a specific field. As with most bibliometric studies, due to the limitation of file formats, only a single electronic database was used, which may introduce some degree of bias.

Second, even with our meticulous vetting and standardization efforts, potential bias might remain from keyword amalgamation and ongoing database revisions. For instance, recent publications may not have had sufficient time to accumulate citations, potentially skewing our citation analysis.

Thirdly, our chosen research time span is from 2014 to 2023. Notably, organ-on-a-chips were first proposed by Michael L. Shuler et al. in the early 21st century, with classic research like the successful construction of a lung organ chip by the Ingber team at Harvard University in 2010 [4,92]. By focusing on publications from 2014 onward, our study might have overlooked some seminal works and contributions made before this period. While we selected this time span based on the exponential growth and advances in the field during this period, we acknowledge the significance of earlier foundational studies.

Finally, we focused solely on literature published in English, implying that relevant research conducted in other languages may have been overlooked. Regardless of these limitations, we believe our findings represent a valid and useful portrayal of the global research landscape surrounding organ-on-a-chip technology, offering unique insights into this burgeoning field of study.

## 5 Conclusions

This investigation offers a comprehensive analysis of global research advancements in the realm of organ-on-a-chip technology. The examination of all relevant English-language literature reveals an encouraging upward trend in research activity. The pivotal contributions come from North America, Asia, and Europe, with the United States taking the lead. Journals with the most organ-on-a-chip related publications include Lab on a Chip, Micromachines and Frontiers in Bioengineering and Biotechnology.

Concerns in this field are largely centered around the utilization of organ-on-a-chip methods for disease modeling, drug discovery, and personalized medicine. The main funding agencies and collaborations are also predominantly found in developed regions, underscoring the need for enhanced cooperation to stimulate the growth of organ-on-a-chip research.

Key research topics that have been identified as current focus areas in this field include metastasis in tumor microenvironments, organoids, tissue engineering, bioprinting and 3D bioprinting, biomaterials, and personalized medicine. Future research directions may maintain focus on these areas and also extend towards the development of complex multi-organ systems, improving the throughput of organ-on-a-chip devices for drug screening, and advancing the mimicry of in vivo conditions.

In sum, we believe this bibliometric study can aid researchers in identifying potential collaboration opportunities and in understanding the knowledge landscape, evolution, and hotspots in the organ-on-a-chip field. This study signals a call to scientists and clinicians alike, emphasizing the potential of organ-on-a-chip technology in transforming biomedical research and healthcare and inviting more attention towards its exploration.

## Funding

This research did not receive any specific grant from funding agencies in the public, commercial, or not-for-profit sectors.

## Data availability statement

Data associated with our study has not been deposited into any publicly available repository. The original contributions presented in the study are included in the article/Supplementary material, and further inquiries can be directed to the corresponding authors.

## CRediT authorship contribution statement

**Chunrong He:** Writing – review & editing, Writing – original draft, Visualization, Validation, Software, Resources, Project administration, Methodology, Investigation, Formal analysis, Data curation, Conceptualization. **Fangfang Lu:** Writing – review & editing, Resources. **Yi Liu:** Writing – review & editing. **Yuanhu Lei:** Writing – review & editing. **Xiaoxu Wang:** Writing – review & editing, Supervision, Funding acquisition. **Ning Tang:** Writing – review & editing, Writing – original draft, Supervision, Methodology, Conceptualization.

## Declaration of competing interest

The authors declare that they have no known competing financial interests or personal relationships that could have appeared to influence the work reported in this paper.

## Figures and Tables

**figs1:**