# Supplementary material for: Emergent trends in organ-on-a-chip applications for investigating metastasis within tumor microenvironment: A comprehensive bibliometric analysis
Source: Heliyon. 2023 Dec 9;10(1):e23504. doi: 10.1016/j.heliyon.2023.e23504 (PMC10770560; doi:10.1016/j.heliyon.2023.e23504)
Supplement: Multimedia component 1 [file mmc1.docx]

**Supplementary Table 1**. The top 100 cited publications in organ-on-a-chip research.

| Rank | Title | First author | Journal | Publication year | Total citations |
| --- | --- | --- | --- | --- | --- |
| 1 | Microfluidic organs-on-chips (DOI:10.1038/nbt.2989) | Bhatia, SN | NAT BIOTECHNOL | 2014 | 1936 |
| 2 | Assembly of functionally integrated human forebrain spheroids (DOI:10.1038/nature22330) | Birey, F | NATURE | 2017 | 644 |
| 3 | Targeting Tumor Microenvironment for Cancer Therapy (DOI:10.3390/ijms20040840) | Roma-Rodrigues, C | INT J MOL SCI | 2019 | 595 |
| 4 | Contributions of microbiome and mechanical deformation to intestinal bacterial overgrowth and inflammation in a human gut-on-a-chip (DOI:10.1073/pnas.1522193112) | Kim, HJ | P NATL ACAD SCI USA | 2016 | 563 |
| 5 | A Review of Cell Adhesion Studies for Biomedical and Biological Applications (DOI:10.3390/ijms160818149) | Khalili, AA | INT J MOL SCI | 2015 | 517 |
| 6 | A four-organ-chip for interconnected long-term co-culture of human intestine, liver, skin and kidney equivalents (DOI:10.1039/c5lc00392j) | Maschmeyer, I | LAB CHIP | 2015 | 516 |
| 7 | Bioprinting 3D microfibrous scaffolds for engineering endothelialized myocardium and heart-on-a-chip (DOI:10.1016/j.biomaterials.2016.09.003) | Zhang, YS | BIOMATERIALS | 2016 | 502 |
| 8 | Advances in organ-on-a-chip engineering (DOI:10.1038/s41578-018-0034-7) | Zhang, BY | NAT REV MATER | 2018 | 500 |
| 9 | Multisensor-integrated organs-on-chips platform for automated and continual in situ monitoring of organoid behaviors (DOI:10.1073/pnas.1612906114) | Zhang, YS | P NATL ACAD SCI USA | 2017 | 471 |
| 10 | Small airway-on-a-chip enables analysis of human lung inflammation and drug responses in vitro (DOI:10.1038/NMETH.3697) | Benam, KH | NAT METHODS | 2016 | 452 |
| 11 | Bioinspired living structural color hydrogels (DOI:10.1126/scirobotics.aar8580) | Fu, FF | SCI ROBOT | 2018 | 404 |
| 12 | Progress in 3D bioprinting technology for tissue/organ regenerative engineering (DOI:10.1016/j.biomaterials.2019.119536) | Matai, I | BIOMATERIALS | 2020 | 392 |
| 13 | Development of a primary human Small Intestine-on-a-Chip using biopsy-derived organoids (DOI:10.1038/s41598-018-21201-7) | Kasendra, M | SCI REP-UK | 2018 | 380 |
| 14 | Biodegradable scaffold with built-in vasculature for organ-on-a-chip engineering and direct surgical anastomosis (DOI:10.1038/NMAT4570) | Zhang, BY | NAT MATER | 2016 | 364 |
| 15 | A liver-on-a-chip platform with bioprinted hepatic spheroids (DOI:10.1088/1758-5090/8/1/014101) | Bhise, NS | BIOFABRICATION | 2016 | 363 |
| 16 | A complex human gut microbiome cultured in an anaerobic intestine-on-a-chip (DOI:10.1038/s41551-019-0397-0) | Jalili-Firoozinezhad, S | NAT BIOMED ENG | 2019 | 361 |
| 17 | Engineered In Vitro Disease Models (DOI:10.1146/annurev-pathol-012414-040418) | Benam, KH | ANNU REV PATHOL-MECH | 2015 | 358 |
| 18 | Organs-on-a-Chip: A Fast Track for Engineered Human Tissues in Drug Development (DOI:10.1016/j.stem.2018.02.011) | Ronaldson-Bouchard, K | CELL STEM CELL | 2018 | 357 |
| 19 | Organoids-on-a-chip (DOI:10.1126/science.aaw7894) | Park, SE | SCIENCE | 2019 | 351 |
| 20 | Microfluidic Organ-on-a-Chip Models of Human Intestine (DOI:10.1016/j.jcmgh.2017.12.010) | Bein, A | CELL MOL GASTROENTER | 2018 | 336 |
| 21 | Microfluidic Blood-Brain Barrier Model Provides In Vivo-Like Barrier Properties for Drug Permeability Screening (DOI:10.1002/bit.26045) | Wang, YI | BIOTECHNOL BIOENG | 2017 | 321 |
| 22 | Human iPSC-based Cardiac Microphysiological System For Drug Screening Applications (DOI:10.1038/srep08883) | Mathur, A | SCI REP-UK | 2015 | 317 |
| 23 | Screening out irrelevant cell-based models of disease (DOI:10.1038/nrd.2016.175) | Horvath, P | NAT REV DRUG DISCOV | 2016 | 307 |
| 24 | Multi-tissue interactions in an integrated three-tissue organ-on-a-chip platform (DOI:10.1038/s41598-017-08879-x) | Skardal, A | SCI REP-UK | 2017 | 306 |
| 25 | Organoid-on-a-chip and body-on-a-chip systems for drug screening and disease modeling (DOI:10.1016/j.drudis.2016.07.003) | Skardal, A | DRUG DISCOV TODAY | 2016 | 302 |
| 26 | Hypoxia-enhanced Blood-Brain Barrier Chip recapitulates human barrier function and shuttling of drugs and antibodies (DOI:10.1038/s41467-019-10588-0) | Park, TE | NAT COMMUN | 2019 | 290 |
| 27 | Organ-on-a-chip: recent breakthroughs and future prospects (DOI:10.1186/s12938-020-0752-0) | Wu, QR | BIOMED ENG ONLINE | 2020 | 285 |
| 28 | Bone marrow-on-a-chip replicates hematopoietic niche physiology in vitro (DOI:10.1038/NMETH.2938) | Torisawa, YS | NAT METHODS | 2014 | 284 |
| 29 | Mature induced-pluripotent-stem-cell-derived human podocytes reconstitute kidney glomerular-capillary-wall function on a chip (DOI:10.1038/s41551-017-0069) | Musah, S | NAT BIOMED ENG | 2017 | 282 |
| 30 | Multi-Organ toxicity demonstration in a functional human in vitro system composed of four organs (DOI:10.1038/srep20030) | Oleaga, C | SCI REP-UK | 2016 | 279 |
| 31 | Reconfigurable microfluidic hanging drop network for multi-tissue interaction and analysis (DOI:10.1038/ncomms5250) | Frey, O | NAT COMMUN | 2014 | 272 |
| 32 | Organ/body-on-a-chip based on microfluidic technology for drug discovery (DOI:10.1016/j.dmpk.2017.11.003) | Kimura, H | DRUG METAB PHARMACOK | 2018 | 245 |
| 33 | Beating heart on a chip: a novel microfluidic platform to generate functional 3D cardiac microtissues (DOI:10.1039/c5lc01356a) | Marsano, A | LAB CHIP | 2016 | 238 |
| 34 | Mobile microrobots for bioengineering applications (DOI:10.1039/c7lc00064b) | Ceylan, H | LAB CHIP | 2017 | 237 |
| 35 | Organ-on-a-chip platforms for studying drug delivery systems (DOI:10.1016/j.jconrel.2014.05.004) | Bhise, NS | J CONTROL RELEASE | 2014 | 237 |
| 36 | Organ-on-a-chip devices advance to market (DOI:10.1039/c6lc01554a) | Zhang, BY | LAB CHIP | 2017 | 236 |
| 37 | 3D microtumors in vitro supported by perfused vascular networks (DOI:10.1038/srep31589) | Sobrino, A | SCI REP-UK | 2016 | 230 |
| 38 | Human Organ Chip Models Recapitulate Orthotopic Lung Cancer Growth, Therapeutic Responses, and Tumor Dormancy In Vitro (DOI:10.1016/j.celrep.2017.09.043) | Hassell, BA | CELL REP | 2017 | 228 |
| 39 | Organs-on-chips with integrated electrodes for trans-epithelial electrical resistance (TEER) measurements of human epithelial barrier function (DOI:10.1039/c7lc00155j) | Henry, OYF | LAB CHIP | 2017 | 222 |
| 40 | A lung-on-a-chip array with an integrated bio-inspired respiration mechanism (DOI:10.1039/c4lc01252f) | Stucki, AO | LAB CHIP | 2015 | 222 |
| 41 | Kidney-on-a-Chip Technology for Drug-Incuced Nephrotoxiciy Screening (DOI:10.1016/j.tibtech.2015.11.001) | Wilmer, MJ | TRENDS BIOTECHNOL | 2016 | 218 |
| 42 | Tubuloids derived from human adult kidney and urine for personalized disease modeling (DOI:10.1038/s41587-019-0048-8) | Schutgens, F | NAT BIOTECHNOL | 2019 | 211 |
| 43 | Three-dimensional brain-on-a-chip with an interstitial level of flow and its application as an in vitro model of Alzheimer's disease (DOI:10.1039/c4lc00962b) | Park, J | LAB CHIP | 2015 | 211 |
| 44 | One-step fabrication of an organ-on-a-chip with spatial heterogeneity using a 3D bioprinting technology (DOI:10.1039/c6lc00450d) | Lee, H | LAB CHIP | 2016 | 208 |
| 45 | Biology-Inspired Microphysiological System Approaches to Solve the Prediction Dilemma of Substance Testing (DOI:10.14573/altex.1603161) | Marx, U | ALTEX-ALTERN ANIM EX | 2016 | 206 |
| 46 | Organ-on-a-Chip Systems: Microengineering to Biomimic Living Systems (DOI:10.1002/smll.201503208) | Zheng, FY | SMALL | 2016 | 196 |
| 47 | A vascularized and perfused organ-on-a-chip platform for large-scale drug screening applications (DOI:10.1039/c6lc01422d) | Phan, DTT | LAB CHIP | 2017 | 193 |
| 48 | The bioprinting roadmap (DOI:10.1088/1758-5090/ab5158) | Sun, W | BIOFABRICATION | 2020 | 192 |
| 49 | Magnetically actuated microrobots as a platform for stem cell transplantation (DOI:10.1126/scirobotics.aav4317) | Jeon, S | SCI ROBOT | 2019 | 190 |
| 50 | Skin-on-a-chip model simulating inflammation, edema and drug-based treatment (DOI:10.1038/srep37471) | Wufuer, M | SCI REP-UK | 2016 | 189 |
| 51 | Body-on-a-chip simulation with gastrointestinal tract and liver tissues suggests that ingested nanoparticles have the potential to cause liver injury (DOI:10.1039/c4lc00371c) | Esch, MB | LAB CHIP | 2014 | 184 |
| 52 | Microfluidic Devices for Drug Delivery Systems and Drug Screening (DOI:10.3390/genes9020103) | Damiati, S | GENES-BASEL | 2018 | 183 |
| 53 | Real-time monitoring of metabolic function in liver-on-chip microdevices tracks the dynamics of mitochondrial dysfunction (DOI:10.1073/pnas.1522556113) | Bavli, D | P NATL ACAD SCI USA | 2016 | 183 |
| 54 | Recent advances in microfluidic technologies for cell-to-cell interaction studies (DOI:10.1039/c7lc00815e) | Rothbauer, M | LAB CHIP | 2018 | 177 |
| 55 | Bio-inspired intelligent structural color materials (DOI:10.1039/c9mh00101h) | Shang, LR | MATER HORIZ | 2019 | 174 |
| 56 | A perfused human blood-brain barrier on-a-chip for high-throughput assessment of barrier function and antibody transport (DOI:10.1186/s12987-018-0108-3) | Wevers, NR | FLUIDS BARRIERS CNS | 2018 | 173 |
| 57 | Organ-On-A-Chip Platforms: A Convergence of Advanced Materials, Cells, and Microscale Technologies (DOI:10.1002/adhm.201700506) | Ahadian, S | ADV HEALTHC MATER | 2018 | 167 |
| 58 | Matched-Comparative Modeling of Normal and Diseased Human Airway Responses Using a Microengineered Breathing Lung Chip (DOI:10.1016/j.cels.2016.10.003) | Benam, KH | CELL SYST | 2016 | 166 |
| 59 | Merging organoid and organ-on-a-chip technology to generate complex multi-layer tissue models in a human retina-on-a-chip platform (DOI:10.7554/eLife.46188) | Achberger, K | ELIFE | 2019 | 164 |
| 60 | 3D bioprinted functional and contractile cardiac tissue constructs (DOI:10.1016/j.actbio.2018.02.007) | Wang, Z | ACTA BIOMATER | 2018 | 157 |
| 61 | Membrane-free culture and real-time barrier integrity assessment of perfused intestinal epithelium tubes (DOI:10.1038/s41467-017-00259-3) | Trietsch, SJ | NAT COMMUN | 2017 | 157 |
| 62 | Human-on-a-chip design strategies and principles for physiologically based pharmacokinetics/pharmacodynamics modeling (DOI:10.1039/c4ib00292j) | Abaci, HE | INTEGR BIOL-UK | 2015 | 157 |
| 63 | Engineering anastomosis between living capillary networks and endothelial cell-lined microfluidic channels (DOI:10.1039/c5lc01050k) | Wang, XL | LAB CHIP | 2016 | 155 |
| 64 | Bioprinting of 3D hydrogels (DOI:10.1039/c5lc90069g) | Stanton, MM | LAB CHIP | 2015 | 153 |
| 65 | State-of-the-Art of 3D Cultures (Organs-on-a-Chip) in Safety Testing and Pathophysiology (DOI:) | Alepee, N | ALTEX-ALTERN ANIM EX | 2014 | 153 |
| 66 | Skin integrated with perfusable vascular channels on a chip (DOI:10.1016/j.biomaterials.2016.11.031) | Mori, N | BIOMATERIALS | 2017 | 152 |
| 67 | Development of a microphysiological model of human kidney proximal tubule function (DOI:10.1016/j.kint.2016.06.011) | Weber, EJ | KIDNEY INT | 2016 | 150 |
| 68 | Design and Demonstration of a Pumpless 14 Compartment Microphysiological System (DOI:10.1002/bit.25989) | Miller, PG | BIOTECHNOL BIOENG | 2016 | 149 |
| 69 | Organs-on-Chips with combined multi-electrode array and transepithelial electrical resistance measurement capabilities (DOI:10.1039/c7lc00412e) | Maoz, BM | LAB CHIP | 2017 | 144 |
| 70 | A microengineered pathophysiological model of early-stage breast cancer (DOI:10.1039/c5lc00514k) | Choi, Y | LAB CHIP | 2015 | 144 |
| 71 | Tumor-on-a-chip platform to investigate progression and drug sensitivity in cell lines and patient-derived organoids (DOI:10.1039/c8lc00596f) | Shirure, VS | LAB CHIP | 2018 | 142 |
| 72 | Microfluidic Organ-on-a-Chip Technology for Advancement of Drug Development and Toxicology (DOI:10.1002/adhm.201500040) | Caplin, JD | ADV HEALTHC MATER | 2015 | 142 |
| 73 | Vascularized cancer on a chip: The effect of perfusion on growth and drug delivery of tumor spheroid (DOI:10.1016/j.biomaterials.2019.119547) | Nashimoto, Y | BIOMATERIALS | 2020 | 140 |
| 74 | A microphysiological model of the human placental barrier (DOI:10.1039/c6lc00259e) | Blundell, C | LAB CHIP | 2016 | 140 |
| 75 | Enhancing the Functional Maturity of Induced Pluripotent Stem Cell-Derived Human Hepatocytes by Controlled Presentation of Cell-Cell Interactions In Vitro (DOI:10.1002/hep.27621) | Berger, DR | HEPATOLOGY | 2015 | 140 |
| 76 | Organs-on-a-chip: a new tool for drug discovery (DOI:10.1517/17460441.2014.886562) | Polini, A | EXPERT OPIN DRUG DIS | 2014 | 140 |
| 77 | Modular, pumpless body-on-a-chip platform for the co-culture of GI tract epithelium and 3D primary liver tissue (DOI:10.1039/c6lc00461j) | Esch, MB | LAB CHIP | 2016 | 139 |
| 78 | Chip-based human liver-intestine and liver-skin co-cultures - A first step toward systemic repeated dose substance testing in vitro (DOI:10.1016/j.ejpb.2015.03.002) | Maschmeyer, I | EUR J PHARM BIOPHARM | 2015 | 139 |
| 79 | Liver 'organ on a chip' (DOI:10.1016/j.yexcr.2017.12.023) | Beckwitt, CH | EXP CELL RES | 2018 | 137 |
| 80 | Long-term maintenance of a microfluidic 3D human liver sinusoid (DOI:10.1002/bit.25700) | Prodanov, L | BIOTECHNOL BIOENG | 2016 | 137 |
| 81 | Aptamer-Based Microfluidic Electrochemical Biosensor for Monitoring Cell-Secreted Trace Cardiac Biomarkers (DOI:10.1021/acs.analchem.6b02028) | Shin, SR | ANAL CHEM | 2016 | 135 |
| 82 | Human organs-on-chips for disease modelling, drug development and personalized medicine (DOI:10.1038/s41576-022-00466-9) | Ingber, DE | NAT REV GENET | 2022 | 134 |
| 83 | A human-airway-on-a-chip for the rapid identification of candidate antiviral therapeutics and prophylactics (DOI:10.1038/s41551-021-00718-9) | Si, LL | NAT BIOMED ENG | 2021 | 133 |
| 84 | Organ-on-a-Chip: A New Paradigm for Drug Development (DOI:10.1016/j.tips.2020.11.009) | Ma, C | TRENDS PHARMACOL SCI | 2021 | 133 |
| 85 | 3D In Vitro Model (R)evolution: Unveiling Tumor-Stroma Interactions (DOI:10.1016/j.trecan.2020.10.009) | Rodrigues, J | TRENDS CANCER | 2021 | 131 |
| 86 | Personalised organs-on-chips: functional testing for precision medicine (DOI:10.1039/c8lc00827b) | van den Berg, A | LAB CHIP | 2019 | 131 |
| 87 | Microfluidic gut-on-a-chip with three-dimensional villi structure (DOI:10.1007/s10544-017-0179-y) | Shim, KY | BIOMED MICRODEVICES | 2017 | 131 |
| 88 | Fabrication and Applications of Microfluidic Devices: A Review (DOI:10.3390/ijms22042011) | Niculescu, AG | INT J MOL SCI | 2021 | 130 |
| 89 | 3D bioprinting for reconstituting the cancer microenvironment (DOI:10.1038/s41698-020-0121-2) | Datta, P | NPJ PRECIS ONCOL | 2020 | 129 |
| 90 | Nanofiber membrane supported lung-on-a-chip microdevice for anti-cancer drug testing (DOI:10.1039/c7lc01224a) | Yang, XY | LAB CHIP | 2018 | 127 |
| 91 | A Human Brain Microphysiological System Derived from Induced Pluripotent Stem Cells to Study Neurological Diseases and Toxicity (DOI:10.14573/altex.1609122) | Pamies, D | ALTEX-ALTERN ANIM EX | 2017 | 127 |
| 92 | On-chip recapitulation of clinical bone marrow toxicities and patient-specific pathophysiology (DOI:10.1038/s41551-019-0495-z) | Chou, DB | NAT BIOMED ENG | 2020 | 125 |
| 93 | Integrated Gut and Liver Microphysiological Systems for Quantitative In Vitro Pharmacokinetic Studies (DOI:10.1208/s12248-017-0122-4) | Tsamandouras, N | AAPS J | 2017 | 125 |
| 94 | Recent Advances in Body-on-a-Chip Systems (DOI:10.1021/acs.analchem.8b05293) | Sung, JH | ANAL CHEM | 2019 | 124 |
| 95 | 3D liver models on a microplatform: well-defined culture, engineering of liver tissue and liver-on-a-chip (DOI:10.1039/c5lc00611b) | No, DY | LAB CHIP | 2015 | 124 |
| 96 | Measuring direct current trans-epithelial electrical resistance in organ-on-a-chip microsystems (DOI:10.1039/c4lc01219d) | Odijk, M | LAB CHIP | 2015 | 122 |
| 97 | Human Gut-On-A-Chip Supports Polarized Infection of Coxsackie B1 Virus In Vitro (DOI:10.1371/journal.pone.0169412) | Villenave, R | PLOS ONE | 2017 | 121 |
| 98 | Organs-on-a-Chip Module: A Review from the Development and Applications Perspective (DOI:10.3390/mi9100536) | Sosa-Hernandez, JE | MICROMACHINES-BASEL | 2018 | 120 |
| 99 | Is it Time for Reviewer 3 to Request Human Organ Chip Experiments Instead of Animal Validation Studies? (DOI:10.1002/advs.202002030) | Ingber, DE | ADV SCI | 2020 | 119 |
| 100 | The Emerging Frontiers and Applications of High-Resolution 3D Printing (DOI:10.3390/mi8040113) | Mao, M | MICROMACHINES-BASEL | 2017 | 119 |
